# Coordinated regulation of timing and strength of synaptic outputs by adrenergic receptors through control of action potentials in Purkinje cells

**DOI:** 10.3389/fncel.2025.1633202

**Published:** 2025-07-22

**Authors:** Kei Furukawa, Shin-ya Kawaguchi

**Affiliations:** ^1^Department of Biophysics, Graduate School of Science, Kyoto University, Kyoto, Japan; ^2^Department of Physiology, Graduate School of Medical and Dental Sciences, Kagoshima University, Kagoshima, Japan

**Keywords:** action potential, axon, Purkinje cell, transmitter release, β-adrenergic receptor

## Abstract

In contrast to conventional view about the faithful signaling in neuronal axons by all-or-none action potentials, recent studies have shown that axons exhibit dynamic change in action potential waveforms and/or conduction velocities in a manner dependent on neuronal activity and/or inputs to axonal compartments from other neurons. It was recently shown that a well-known second messenger cAMP negatively regulates the axonal voltage-gated Na^+^ channels, which decreases the amplitude and conduction velocity of action potentials in axons of cerebellar Purkinje cells. To understand the signaling mechanism and physiological context of the cAMP-mediated action potential modulation, we studied the involvement of one of neuromodulators, adrenergic system, using direct patch-clamp recordings from axons and/or terminals of Purkinje cells. We demonstrate that Purkinje cell axons exhibit negative control of action potentials in amplitude and conduction velocity by β-adrenergic receptors in a manner dependent on the axonal length through specific reduction of axonal Na^+^ currents. On the other hand, β-adrenergic receptors increased presynaptic release probability without changing the amount of readily releasable vesicles in axon terminals of Purkinje cells. Together, our data highlight a physiological pathway to activate cAMP signaling to cause the axonal length-dependent dynamic changes in the timing and strength of synaptic transmission.

## 1 Introduction

Action potential (AP) propagation in a long axon is essential for rapid and reliable information transfer in the nervous system. It is well-known that APs propagate in an axon faithfully as digital all-or-none signals at an identical velocity keeping its waveform constant from the site of initiation near the soma to the distal axon terminals. In spite of such a classical dogma, recent findings exhibit the analogue capabilities of axonal signaling, in which the amplitude, time course, and/or conduction velocity of an AP can be modulated in various cases, according to neuronal activity and/or intracellular molecular signaling (Shu et al., 2006; Debanne et al., 2011; Zbili and Debanne, 2019; Byczkowicz et al., 2019; Lezmy et al., 2021). Such modification of APs might affect transmitter release via changing presynaptic Ca^2+^ influx. Indeed, attenuation of an AP in velocity and amplitude by cytosolic cAMP, a well-known second messenger, results in changes in timing and strength of axonal outputs in a graded manner dependent on the length of axons in cerebellar Purkinje cells (PCs) (Furukawa et al., 2024; Abate et al., 2024). However, the upstream signaling pathway for cAMP underlying the dynamic control of axonal outputs remains elusive.

In this study, we focused on β-adrenergic receptors (β-AR), coupled to Gs-type trimeric G protein, which activates adenylyl cyclase and increases intracellular cAMP. Indeed, the axons releasing norepinephrine (NE), an endogenous ligand for β-AR, originating from the locus ceruleus, project to the whole cerebellum (Stanley et al., 2023). Further, in the cerebellum, NE was shown to modulate the velocity of AP conduction in parallel fibers (Byczkowicz et al., 2019), and also the presynaptic release at axon terminals of various cell types, such as parallel and climbing fibers, and GABAergic interneurons, as well as in hippocampal mossy fibers and calyx of Held synapses in the auditory pathway (Llano and Gerschenfeld, 1993; Huang and Kandel, 1996; Kondo and Marty, 1998; Leão and von Gersdorff, 2002; Saitow et al., 2005; Hirono and Obata, 2006; Carey and Regehr, 2009; Martín et al., 2020). Thus, it would be possible that adrenergic inputs also work on PC axons, thereby controlling the timing and strength of signal outputs of cerebellar cortical circuits depending on the animals’ awake state. However, because of the technical hurdle to directly evaluate axonal AP propagation and presynaptic release probability, functional impacts of adrenergic inputs on the PC axonal signaling remains unclear.

Here, taking advantages of direct patch-clamp recordings from PC axons and terminals in primary culture (Kawaguchi and Sakaba, 2015; Furukawa et al., 2024), we examined whether and how β-AR plays a role in the AP conduction and presynaptic release. Direct patch-clamp recordings from intact long axons showed that β-AR attenuates the AP conduction in velocity and amplitude through reduction of axonal Na^+^ currents. We also biophysically analyzed the direct effect of β-AR activation on transmitter release, and obtained data showing increase in the release probability, but not the size of readily releasable pool of vesicles in PC axon terminals.

## 2 Materials and methods

### 2.1 Animals

All experimental procedures were conducted in accordance with regulations on animal experimentation in Kyoto University and approved by the local committee for animal experiments in Graduate School of Science, Kyoto University (#202412). In this study, Wistar rats (Slc:Wistar, Japan SLC Inc.) of either sex were used.

### 2.2 Preparation of cerebellar primary cultures

The method for preparing primary dissociated cultures of cerebellar neurons was similar to that in a previous study (Kawaguchi and Sakaba, 2015). Briefly, cerebella were dissected out from newborn rats and their meninges were removed. The cerebella were incubated at 37°C in Ca^2+^ and Mg^2+^-free Hank’s balanced salt solution containing 0.1% trypsin and 0.05% DNase for 15 min. Cells were dissociated by trituration and seeded on poly-D-lysine-coated cover slips in Dulbecco’s modified Eagle’s medium: nutrient mixture F12-based medium containing 2% fetal bovine serum. One day after seeding, ∼80% of the medium was replaced by basal medium eagle (BME)-based medium. Thereafter, about half of the medium was changed every 3–4 days with fresh BME-based medium together with cytosine arabinoside (4 μM) to inhibit proliferation of glial cells. At 4–5 days after seeding, PCs were transfected with EGFP by an AAV vector serotype 2 under the control of CA promoter (AAV2-CA-EGFP). PCs were visually identified by their large cell bodies and thick dendrites. Experiments were performed > 21 days after seeding.

### 2.3 Electrophysiology

Electrophysiological experimental procedures were similar to that in a previous study (Furukawa et al., 2024). Patch-clamp recordings were performed with an amplifier (EPC10, HEKA) mounted on an inverted microscope (IX71, Olympus) equipped with a 40×, 0.95 numerical aperture (NA) objective at room temperature (20–24°C), in an extracellular solution containing the following (in mM): 145 NaCl, 10 HEPES, 10 D-glucose, 2 CaCl_2_, 1 MgCl_2_, pH 7.3–7.4 adjusted by KOH, and osmolarity 300–320 mOsm/kgH_2_O. Images were obtained with a sCMOS camera (Zyla4.2, Andor). In some experiments, 2,3-dioxo-6-nitro-1,2,3,4-tetrahydrobenzo[f]quinoxaline-7-sulfonamide (NBQX, 10 μM), picrotoxin (50 μM), and tetrodotoxin (TTX, 1 μM) were applied to the extracellular solution to inhibit glutamatergic EPSCs, GABAergic IPSCs, and APs, respectively. For cell-attached recordings, patch pipettes were filled with the extracellular solution. For current-clamp recordings, K-gluconate-based internal solution with the following composition (mM) was used: 155 K-gluconate, 7 KCl, 10 HEPES, 0.5 ethylene glycol bis (β-aminoethylether) N,N,N′,N′-tetraacetic acid (EGTA), 2 Mg-ATP, 0.2 Na-GTP, pH 7.3–7.4 adjusted by KOH, and osmolarity 310–340 mOsm/kgH_2_O. For voltage-clamp recordings from PCs’ target postsynaptic neurons, patch pipettes were filled with CsCl-based internal solution containing the following (in mM): 170 CsCl (or 137 CsCl and 33 Cs-gluconate), 10 HEPES, 5 EGTA, 2 Mg-ATP, 0.2 Na-GTP, pH 7.3–7.4 adjusted by CsOH, and osmolarity 310–340 mOsm/kgH_2_O. For direct recordings from PC axon terminals, CsCl-based internal solution containing 0.5 mM EGTA was used in the presence of external TTX and tetraethylammonium (TEA, 2 mM). For measurements of voltage-gated Na^+^ and K^+^ currents, K-gluconate-based internal solution was used, with correction of the liquid junction potential (∼15 mV). To activate β-adrenergic receptors, isoproterenol (ISO) was added to the bath at a relatively high concentration (100 μM) as in Martín et al., 2020, taking it into account that the direct patch-camp recordings from small size of axonal compartments are technically demanding and the success rate of recordings is low.

Membrane potential of the PCs were held at −70 mV unless otherwise specified. Evoked IPSCs (eIPSCs) were recorded from PCs’ target neurons under the voltage-clamp at −70 to −120 mV to avoid unclamped voltage-gated Na^+^ currents. Series resistances at the PC soma, axon, and terminal (in MΩ) were 12 ± 4, 72 ± 30, and 190 ± 76 (mean ± SD; *n* = 17, 13, and 19 cells), and were compensated online by 40%–60%. Online compensation (20%–60%) for series resistance was applied for IPSC recordings from PCs’ target cells. APs were elicited by current injection of 0.5–1 nA for 10 ms into PC soma and those with intervals > 50 ms were accepted for analysis to avoid the possible modulation of APs by high frequency firing (Kawaguchi and Sakaba, 2015). eIPSCs were used for analysis unless the failure rate excessively changed during recordings. Membrane capacitance (C_*m*_) was measured by sine +DC technique (Neher and Marty, 1982) implemented on Patchmaster software (HEKA), in which presynaptic terminals were held at −80 mV and the sine wave (1 kHz and the peak amplitude of 30 mV) was applied on the holding potential. As membrane conductance fluctuates for tens of ms after the depolarizing pulse due to large change in presynaptic conductance caused by depolarization, C_*m*_ was usually measured ∼50 ms after the depolarization. Data were digitized and sampled at 20–100 kHz, and low-pass filtered at 3 kHz.

### 2.4 Analysis

All obtained data were analyzed using Patchmaster and Igor Pro (WaveMetrics). APs, voltage-gated currents, and eIPSCs were detected using TaroTools extensions^1^ implemented on Igor Pro. To measure the distance from the soma to the recording site, ImageJ (NIH) was used. The onset of eIPSC was defined as the first time point at which the recorded current value exhibited change larger than 2SD of that at the basal condition. In some analysis, the onset timing of APs was assessed by the peak of the 2*^nd^*-derivative of membrane potentials.

The current (I_Na^+_)-voltage (Vm) relation for voltage-gated Na^+^ currents was fitted by the following equation based on the Boltzmann function:


(1) $$I_{N\,a^{+}}=G_{N\,a^{+}\,m\,a\,x}\cdot \frac{1}{1+e^{\frac{V_{1/2}-V_{m}}{k}}}\cdot \left(V_{m}-E_{N\,a^{+}}\right)+G_{l\,e\,a\,k}$$

where E_Na^+_ is the equilibrium potential for Na^+^. Fitting of current-voltage relationship for each axonal I_Na^+_ yielded three important parameters for the activation of voltage-gated Na^+^ channels: the relative peak conductance (G_Na^+max_) and the voltage for half-maximal activation of Na^+^ (V_1/2_), and the slop factor (k).

To estimate the voltage-clamped area in a direct recording from a presynaptic terminal, capacitive transients in response to hyperpolarizing pulses (5–20 mV) were used. The capacitive transient at a terminal followed a single exponential function with a time constant 0.24 ± 0.06 ms (mean ± SD), so that the clamped membrane area of the terminal (and neighboring axon) was estimated to be 1.5 ± 0.9 pF on average (mean ± SD). Considering the correlation between the clamped area size and the presynaptic Ca^2+^ currents (I_Ca^2+_) or C_*m*_ increase (Furukawa et al., 2024), I_Ca^2+_ and C_*m*_ increase were normalized by the clamped membrane capacitance of individual boutons.

### 2.5 Statistics

Data in all figures are presented as mean ± SEM unless otherwise mentioned. The difference between groups was evaluated by Wilcoxon signed-rank test for paired groups or Mann–Whitney U test for unpaired ones. Two-way analysis of variance (ANOVA) was also used to evaluate data shown in Figure 3B. Spearman’s correlation coefficients were used. Statistical significance was considered to be *p* < 0.05.

## 3 Results

### 3.1 Modulation of synaptic outputs from PCs by β-AR

**FIGURE 1:**
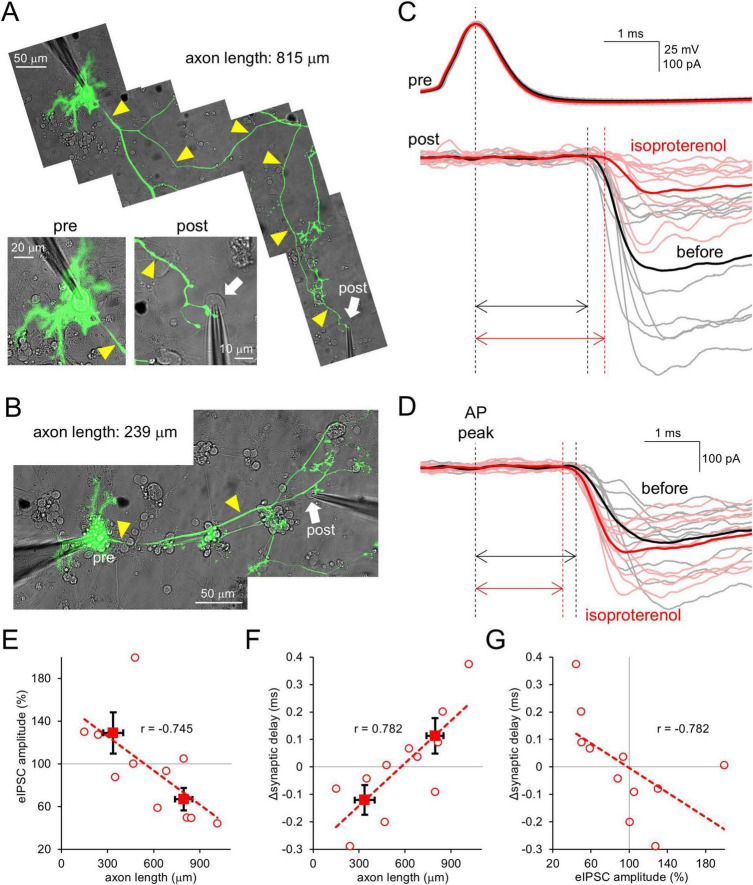
β-AR modulates strength and timing of PC outputs depending on axonal length. **(A,B)** Paired patch-clamp recordings from the PC soma and its target postsynaptic neuron connected by a long **(A)** or short **(B)** axon (yellow arrowheads). **(C,D)** Representative traces for APs and eIPSCs (gray, pink) and their averages (black, red) recorded from the synaptically-connected neurons [shown in **(A,B)**] before and after the isoproterenol application. Traces were time-aligned by the peak of presynaptic APs. Double arrows indicate synaptic delays. **(E,F)** eIPSC amplitude **(E)** and change in synaptic delay **(F)** after the isoproterenol application plotted as a function of axonal length connecting the cell pairs. Spearman’s correlation coefficient *r* = –0.745, 0.01 < *p* < 0.02 **(E)** and *r* = 0.782, 0.005 < *p* < 0.01 **(F)**. **(G)** Change of synaptic delay after the isoproterenol application plotted as a function of relative eIPSC amplitude. *r* = –0.782, 0.005 < *p* < 0.01. Linear fits to data points are presented by dotted lines. Data for individual pairs (open circles) and mean ± SEM (closed squares) are shown. *n* = 11 pairs.

First, we examined how β-AR activation affects synaptic outputs from PCs in cerebellar culture. A cultured PC keeps an intact long axon (sometimes > 1 mm) from the soma to a lot of terminals. PCs were EGFP-labelled using an AAV vector, and simultaneous patch-clamp recordings were performed from the soma and a postsynaptic neuron synaptically connected from EGFP-positive axon varicosities (Figures 1A, B). The PC soma was current-clamped, and APs were elicited by current injection (0.5–1 nA, 10 ms). Then, evoked IPSC (eIPSC) was recorded from the voltage-clamped PCs target neuron (Figures 1C, D). Isoproterenol (ISO), a β-AR agonist, was applied to the extracellular bath to activate β-AR. In line with a previous report showing dynamic change of eIPSCs by the cAMP increase upon extracellular application of forskolin (Furukawa et al., 2024), eIPSC amplitude changed after the ISO application, in a manner dependent on the axonal length between two recorded cells (< 500 μm, 129 ± 19%; > 500 μm, 67 ± 11%, *p* < 0.05; Figures 1C–E). In addition, the reduction of eIPSC amplitude was accompanied with an increase in synaptic delay (*r* = −0.782, *p* < 0.01; Figures 1C, D, F, G and Supplementary Figure 1A). Thus, these data suggest that β-AR, which is expected to increase intracellular cAMP, modulates the strength and timing of PCs’ outputs with a negative relation to the axonal length, in line with previous works (Furukawa et al., 2024).

### 3.2 AP attenuation in PC axons by β-AR

**FIGURE 2:**
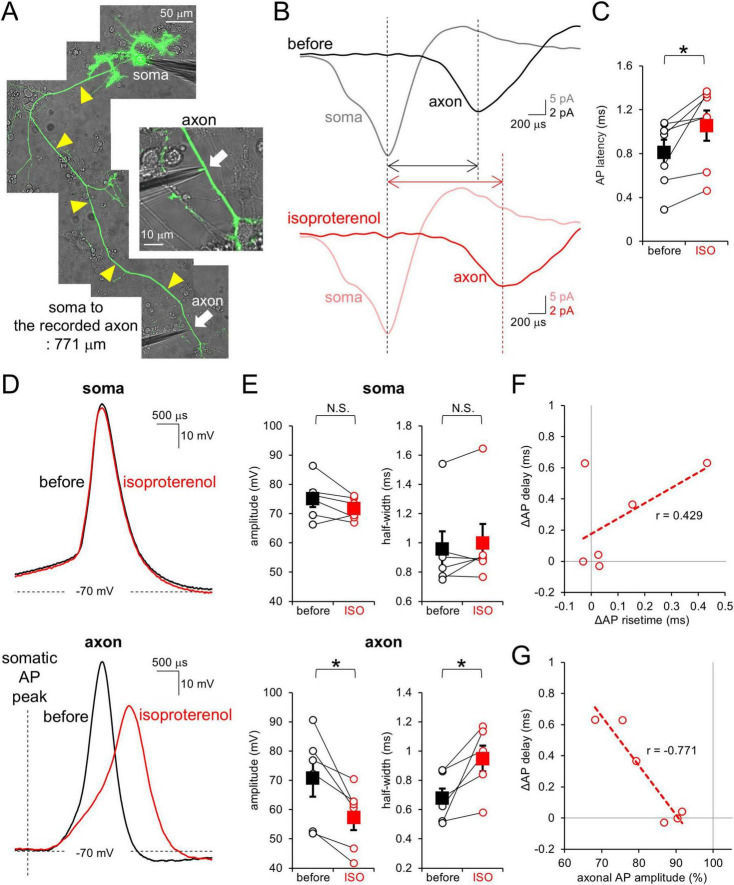
β-AR attenuates AP in conduction velocity and amplitude at PC axons. **(A)** Image of simultaneous cell-attached recordings from the PC soma and its axon. Yellow arrowheads indicate the axon. **(B)** Representative spontaneous APs recorded from the soma (gray, pink) and axon (black, red) before (top) and after (bottom) the isoproterenol application. Traces were time-aligned by the peak timing of somatic AP. **(C)** Axonal AP latency from the somatic AP before and after the isoproterenol (ISO) application. Data for individual pairs (open circles) and mean ± SEM (closed squares) are shown. *n* = 7 pairs. *p < 0.05. **(D)** Representative traces for somatic (top) and axonal (bottom) APs before (black) and after (red) the isoproterenol application. **(E)** Amplitude (left) and half-width (right) of somatic and axonal APs before and after the isoproterenol (ISO) application. Data for individual recording sites (open circles) and mean ± SEM (filled squares) are shown. **(F,G)** Isoproterenol-mediated changes in the axonal AP latency from the somatic one plotted as a function of change in risetime **(F)** or amplitude **(G)** of axonal AP. *r* = 0.429, 0.2 < *p* < 0.5 **(F)** and *r* = –0.771, 0.1 < *p* < 0.2 **(G)**. Linear fits to data points are presented by dotted lines. *n* = 6 pairs.

We next explored the mechanism by which synaptic outputs were modulated by β-AR activation in a manner dependent on the axonal distance. Previous studies showed that cAMP attenuated axonal AP conduction in terms of velocity and amplitude, leading to axonal length-dependent modulation of synaptic outputs in PCs (Furukawa et al., 2024). To test whether β-AR plays a role in the modulation of AP conduction in PC axons, paired cell-attached recordings from the PC soma and its axon were performed (Figure 2A). As shown in Figure 2B, a spontaneous AP was observed first at the soma, later at the axon. ISO significantly increased this latency (0.81 ± 0.12 ms to 1.06 ± 0.14 ms at 485 ± 60 μm away from the soma, *p* < 0.05; Figure 2C and Supplementary Figure 1B), suggesting that β-AR slows the AP conduction velocity in PC axons.

To obtain an insight into a mechanism underlying the apparent slowing of AP conduction, we next focused on the waveform of an AP. The PC soma and axon were current-clamped so that the membrane potentials were kept around −70 mV, and APs were evoked by current injection into the soma. While somatic APs were little affected by the ISO application (amplitude: 96 ± 2%; half-width: 105 ± 4%), axonal APs conducting from the soma were attenuated by ISO in amplitude and slowed in time course (amplitude: 82 ± 4%; half-width: 144 ± 17% at 556 ± 51 μm away from the soma; Figures 2D, E and Supplementary Figure 1C). The ISO-mediated increase in AP latency between recording sites at the soma and an axon showed only a marginal dependency on the changes in time for an AP peaking from its onset (Figure 2F). In contrast, the altered AP latency showed a close relation to the reduction of AP amplitude (∼0.3 ms delay per ∼10% reduction of amplitude; Figure 2G). Thus, attenuated size of APs seems to slow the AP conduction in PC axons, with slight contribution of slowed waveforms of axonal APs to the increased latency of AP peaks between two recording sites. Taken together, our results indicate that β-AR attenuates axonal AP waveform, but not somatic AP, coordinately reducing its conduction velocity, in a similar manner to the effect of cAMP shown in previous studies (Furukawa et al., 2024).

### 3.3 Reduction of axonal Na^+^ current in PCs by β-AR

**FIGURE 3:**
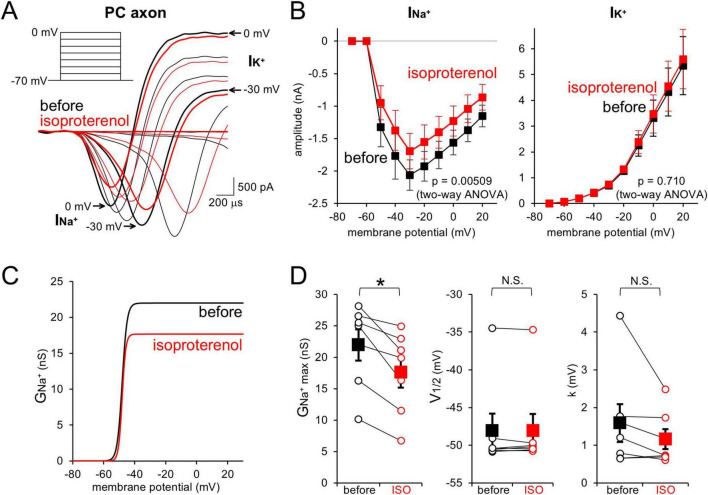
β-AR decreases PC axonal Na^+^ currents. **(A,B)** Representative traces **(A)** and current-voltage (I–V) relations **(B)** for voltage-gated Na_+_ (I_Na^+_) and K^+^ currents (I_K^+_) upon depolarization pulses [**(A)**, inset] recorded from a PC axon before (black) and after (red) the isoproterenol application. *n* = 7 axons. **(C,D)** Voltage-dependent activation profile of axonal Na^+^ conductance **(C)** and three parameters **(D)**: relative peak conductance (G_Na^+max_, left), voltage for half-maximal activation (V_1/2_, middle), and slop factor (k, right) before and after the isoproterenol (ISO) application. Data for individual axons (open circles) and mean ± SEM (filled squares) are shown. *p < 0.05.

Previously, we showed that direct activation of cAMP pathway in PC axons reduces voltage-dependent Na^+^ currents (Furukawa et al., 2024). To examine whether the Na^+^ current reduction underlies the AP attenuation by β-AR (see Figures 2), PC axonal trunk was voltage-clamped, and voltage-gated Na^+^ and K^+^ currents (INa^+^ and IK^+^) upon step depolarizations (from −70 mV) were recorded. ISO decreased the amplitude of INa^+^ (−1.93 ± 0.23 nA to −1.55 ± 0.26 nA at −20 mV, *p* < 0.05) without changing the voltage-dependency, but did not affect IK^+^ (3.31 ± 0.70 nA to 3.48 ± 0.74 nA at 0 mV, *p* > 0.05; Figure 3). Thus, β-AR specifically reduces I_Na^+_ in the axon, decreasing the membrane excitability dependent in principle on the ratio of Na^+^ influx to K^+^ efflux, which would be responsible for the AP attenuation demonstrated above, through activating signal pathway including cAMP.

### 3.4 Augmentation of transmitter release in PC axon terminals by β-AR

**FIGURE 4:**
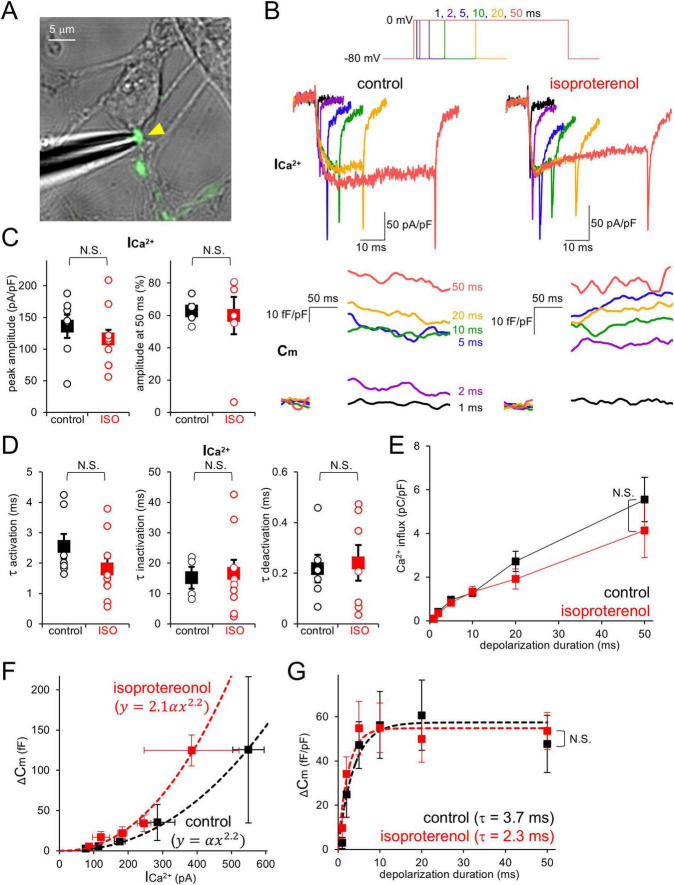
β-AR increases presynaptic release probability without changing Ca^2+^ currents and RRP size. **(A)** Direct patch-clamp recording from a PC axon terminal (yellow arrowhead). **(B)** Representative presynaptic Ca^2+^ currents (I_Ca^2+_) and membrane capacitance (Cm) increase upon presynaptic depolarization pulses (1–50 ms, top) in the absence (left, control) or presence (right) of extracellular isoproterenol. **(C)** I_Ca^2+_ amplitude at the peak (left) during depolarization to 0 mV and relative amplitude at 50 ms normalized by the peak (right) in the absence or presence of isoproterenol (ISO). **(D)** Time constants for activation (left), inactivation (middle), and deactivation (right) of I_Ca^2+_ in the absence or presence of ISO. **(E)** Total charge for Ca^2+^ influx plotted as a function of depolarization pulse duration in the absence or presence of ISO. **(F,G)** C_m_ increase plotted as a function of I_Ca^2+_ amplitude (**F**, upon 1–2 ms depolarization) or duration of depolarization pulse **(G)**. Fits to data points [the 2.2th power dependency on I_Ca^2+_ for **(F)** or the exponential curve for **(G)**] are indicated as dotted lines. In **(B,C,E,G)**, Ca^2+^ current and charge, and Cm are normalized by the size of voltage-clamped area. *n* = 7 (control) and 12 (isoproterenol) boutons.

Finally, we examined whether β-AR increases presynaptic release probability in PC axon terminals, as has been demonstrated upon cAMP increase in various synapses including PC terminals (Capogna et al., 1995; Chavez-Noriega and Stevens, 1994; Kaneko and Takahashi, 2004; Meadows et al., 2021; Salin et al., 1996; Weisskopf et al., 1994; Furukawa et al., 2024). Taking advantage of direct patch-clamp recordings from PC boutons, depolarizing pulses (−80 mV to 0 mV, 1–50 ms) were applied to the voltage-clamped terminal in the presence of TTX, and presynaptic I_Ca^2+_ and subsequent increase in membrane capacitance (Cm) at a terminal were recorded (Figures 4A, B). The amplitude, extent of inactivation, kinetics of activation and deactivation of I_Ca^2+_ showed substantial variability in different boutons, but were in average not affected by the external ISO (Figures 4C–E). On the other hand, Cm tended to more efficiently increase in relation to the I_Ca^2+_ amplitude in the presence of ISO (∼2 fold of control, upon 1–2 ms depolarization; Figure 4F), although reaching a similar maximum level compared to control condition upon longer presynaptic depolarization (control: 47.7 ± 13.0 fF/pF; ISO: 53.6 ± 8.3 fF/pF; *p* > 0.05; upon 50 ms depolarization; Figure 4G). Together, our data suggest that β-AR facilitates transmitter release from PC axon terminals, without changing the total amount of synaptic vesicles categorized into the readily releasable pool (RRP), which would be responsible for the facilitation of synaptic outputs at short axonal length as shown in Figure 1E. Thus, the action of β-AR on synaptic outputs nicely matched a previous study for the action of intracellular cAMP on release in PC boutons (Furukawa et al., 2024).

## 4 Discussion

In this study, taking advantages of direct patch-clamp recordings from intact long axons/terminals of cultured PCs, we unveiled that β-AR makes axonal AP waveforms smaller and slower and also lowers its conduction velocity through specifically reducing axonal Na^+^ currents. Besides, β-AR has another direct effect at axon terminals by which presynaptic transmitter release is facilitated. These two opposing actions of β-AR on axonal outputs, reduction of presynaptic Ca^2+^ influx by the AP attenuation and increase in the release probability, together make it possible to bidirectionally regulate the timing and strength of synaptic outputs from PC terminals depending on the axonal length.

### 4.1 Analogue modulation of AP conduction

Shapes of APs arriving at axon terminals decide the opening of the presynaptic voltage-gated Ca^2+^ channel (Cav), thereby giving an impact on the transmitter release, although the extent is variable depending on the cell type (Sabatini and Regehr, 1997; Borst and Sakmann, 1999; Geiger and Jonas, 2000; Taschenberger and von Gersdorff, 2000; Boudkkazi et al., 2011; Kawaguchi and Sakaba, 2015; Zbili et al., 2020). When the opening kinetics of presynaptic Cav is fast enough relative to the time course of AP waveform, a majority of Cav has a chance to get activated upon a single AP even if the peak amplitude of AP is somehow altered (Borst and Sakmann, 1998; Bischofberger et al., 2002). In such a case, Ca^2+^ influx is predominantly determined by the time course of AP in decay phase, during which Ca^2+^ influx gradually increases as the driving force gets larger due to repolarization. Indeed, presynaptic terminals of calyx of Held synapse or hippocampal mossy fibers exhibit more powerful effects of the time course rather than the amplitude of APs on Ca^2+^ influx and the resultant transmitter release (Geiger and Jonas, 2000; Bischofberger et al., 2002). On the other hand, only a limited population of Cav opens in the case when the presynaptic AP is rapid compared to the Cav activation, giving rise to larger sensitivity of Ca^2+^ influx to the AP amplitude. Indeed, change in the AP amplitude has been shown to control the Ca^2+^ influx in boutons of cerebellar granule cells, GABAergic interneurons, and PCs (Kawaguchi and Sakaba, 2015; Kawaguchi and Sakaba, 2017; Trigo and Kawaguchi, 2023; Furukawa et al., 2024). Particularly at synapses such as PCs which are functionally designed to undergo transmitter release based on the tight coupling between Cav and release machinery, the number of Cav activated, rather than the total amount of Ca^2+^ entering into the cytoplasm, mainly determines the total amount of vesicles undergoing exocytosis (Díaz-Rojas et al., 2015). In this study, decreased AP amplitude by β-AR (see Figure 2) would negatively regulate presynaptic Ca^2+^ influx and subsequent transmitter release, as shown in previous studies (see Figure 1; Kawaguchi and Sakaba, 2015; Díaz-Rojas et al., 2015; Furukawa et al., 2024). Thus, AP waveform and the resultant Ca^2+^ influx dynamically control synaptic outputs based on an elaborate presynaptic design, which would decide whether and to what extent the AP modulation impacts the intensity of axonal outputs.

It has been reported that the AP conduction velocity at cerebellar parallel fibers is speeded by NE, although the direction of modulation is opposite between axons of granule cells and PCs (see Figure 2; Byczkowicz et al., 2019). At parallel fibers, NE increases membrane excitability by activating HCN channels via activation of β-AR at physiological temperature (35°C). On the other hand, here we demonstrated that β-AR reduces axonal Na^+^ currents in PCs at room temperature (Figure 3), in line with the effect of internal cAMP decreasing Na^+^ currents, but not activating HCN channels, which results in decreased membrane excitability (Furukawa et al., 2024). It would be an important issue to be clarified in a future whether the negative control of APs in PC axons is the case also at the physiological temperature. The discrepancy between parallel fibers and PC axons would be ascribed to selective expression of cAMP-insensitive HCN1 in PCs (Wang et al., 2001; Notomi and Shigemoto, 2004). As shown in Figure 3C, β-AR decreases the voltage-dependent activation of Na^+^ conductance without changing the voltage-dependency. It remains an open question whether β-AR decreases the activation of Nav channels for example by reducing single channel conductance or open probability, or decreases the fraction of available Na^+^ channels by increasing inactivated fraction at −70 mV. We could not detect any changes in voltage-dependent axonal K^+^ currents (Figure 3), but more detailed analysis of Na^+^ and K^+^ currents is preferred for accurate evaluation, for example with pharmacological isolation. In cortical pyramidal cells, voltage-gated Na^+^ channels at an axon initial segment (AIS) are negatively regulated by 5-HT1A receptors, coupled to Gi/o-type of G protein which inhibits adenylyl cyclase (Yin et al., 2017), in contrast to the lack of changes of somatic Na^+^ currents upon cAMP increase in PCs (Furukawa et al., 2024). Nav1.2 is expressed at the AIS of pyramidal cells, while PCs are reported to express Nav1.1, 1.4, 1.6, 1.7, 1.8, and 1.9, but not Nav1.2 (Schaller and Caldwell, 2003; Lein et al., 2007). On the other hand, altered voltage-gated K^+^ currents by dopaminergic receptors (Yang et al., 2013), and change in the AP conduction velocity or waveform by other neurotransmitters such as 5-HT, and/or adenosine were also suggested in a variety of neuronal axons such as cerebellar granule cells, and pyramidal cells in the cortex and hippocampus (Sasaki et al., 2011; Byczkowicz et al., 2019; Lezmy et al., 2021). Various receptors and subsequent signaling pathways activated by those transmitters, provide patterns of modulation of AP conduction in distinct types of neurons. Moreover, axonal local receptors for glutamate or GABA also control AP waveforms or firings (Trigo et al., 2010; Sasaki et al., 2011; Zorrilla de San Martin et al., 2017).

### 4.2 Facilitation of presynaptic transmitter release by β-AR

It is well-known that cAMP is an important regulator of presynaptic function, for example inducing presynaptic long-term potentiation by which transmitter release is augmented (Chavez-Noriega and Stevens, 1994; Weisskopf et al., 1994; Capogna et al., 1995; Salin et al., 1996; Kaneko and Takahashi, 2004; Meadows et al., 2021). Thus, the upstream and downstream signaling mechanism of cAMP potentiating transmitter release has been one of the most important issues. In various types of neurons, NE and β-AR are the typical upstream factors leading to cAMP increase (Llano and Gerschenfeld, 1993; Huang et al., 1996; Huang and Kandel, 1996; Kondo and Marty, 1998; Saitow et al., 2005; Martín et al., 2020). Recent works on the cerebellar parallel fiber boutons and synaptosome of cortical neurons using immunoelectron microscopy and/or transgenic mice showed that β-AR-caused cAMP elevation increases the total amount of synaptic vesicles belonging to the readily releasable pool (RRP), through Epac-mediated modulation of RIM1 and Munc-13 at active zones, resulting in potentiation of release (Ferrero et al., 2013; Martín et al., 2020). In contrast, RRP size was little altered by ISO in PC boutons (see Figure 4). A recent study, performing direct patch-clamp recordings and fluorescent imaging of vesicular fusion at PC terminals, suggests that the Ca^2+^ influx through Cav channels only activates limited vesicles which are located very close to Cav among total releasable ones (Inoshita and Kawaguchi, 2025), presumably due to potent and rapid cytosolic Ca^2+^ buffering mediated by abundant calbindin (Fierro and Llano, 1996). Such functional design of PC boutons minimizes the efficiency of increasing total releasable vesicles for augmenting release. Rather, as shown in Figure 4F, β-AR augments release by increasing Ca^2+^ sensitivity, without affecting neither Cav currents nor RRP size, in line with findings of augmentation of release by cAMP at calyx of Held synapses and PC boutons (Yao and Sakaba, 2010; Furukawa et al., 2024). A previous study using direct bouton recordings coupled with Ca^2+^ uncaging showed that the rate of transmitter release in PCs gets faster in a manner dependent on intracellular Ca^2+^ (Kawaguchi and Sakaba, 2015). Thus, the decreased synaptic delay in short axonal-outputs of PCs (shown in Figure 1F) might be ascribed to the β-AR-caused heightened Ca^2+^ sensitivity for release.

The modulation of PC axonal APs and outputs by adrenergic inputs demonstrated here, casts further questions which should be addressed in a future. For example, while adrenergic axons innervate the whole cerebellum (Stanley et al., 2023), subcellular localization and abundance of β-AR in PCs (i.e., axonal site and/or somatodendritic compartments) remain unclear. Considering the fact that the β-AR-mediated negative regulation of APs and synaptic outputs was evident when the bouton was more distant from the soma (see Figures 1, 2), the β-AR located at axons and terminals would be involved. Studying whether the effect of ISO is abolished by an antagonist for β-AR, as well as how long the β-AR-mediated modulation lasts, for example by studying the reversibility after washing the agonist, will further highlight the critical role of adrenergic inputs in axonal signaling of PCs. In addition, α1- and/or α2- adrenergic receptors (coupled to Gq/11 and Gi/o proteins, respectively) are also reported to modulate transmitter release (Leão and von Gersdorff, 2002; Hirono and Obata, 2006; Carey and Regehr, 2009). Thus, it would be possible for NE to activate various combinations of α- and β-ARs, possibly providing wide range of modulation of synaptic outputs, which should be addressed in future studies.

## Data Availability

The original contributions presented in this study are included in this article/Supplementary material, further inquiries can be directed to the corresponding author.

## Footnotes

1
https://sites.google.com/site/tarotoolsregister/
