# Supplementary material for: Coordinated regulation of timing and strength of synaptic outputs by adrenergic receptors through control of action potentials in Purkinje cells
Source: Front Cell Neurosci. 2025 Jul 22;19:1633202. doi: 10.3389/fncel.2025.1633202 (PMC12321777; doi:10.3389/fncel.2025.1633202)
Supplement: Supplementary file 1 [file Image_1.pdf]

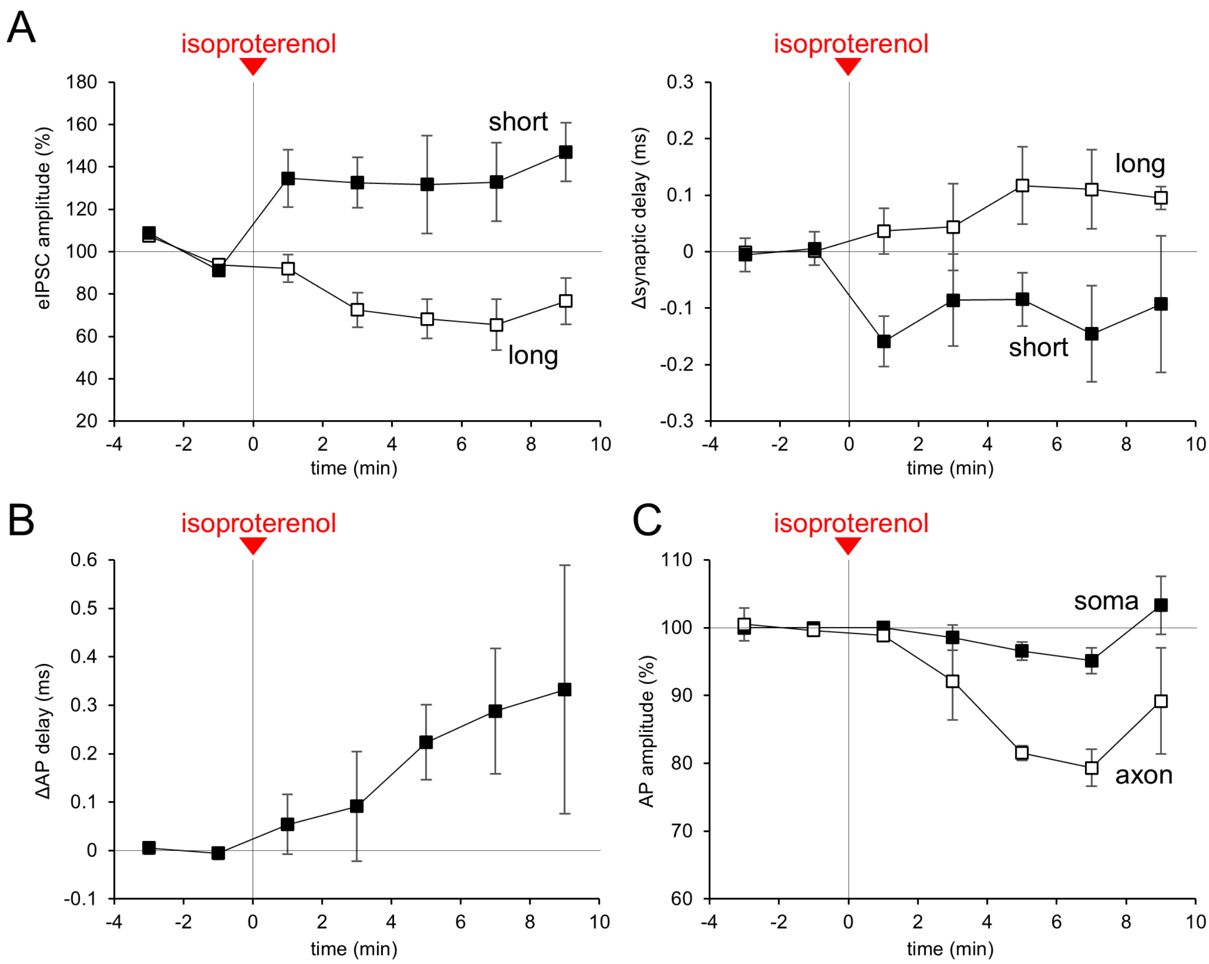

Supplementary Figure S1. Time courses of changes in synaptic transmission and APs in Purkinje cells before and after the isoproterenol application.

A, Normalized eIPSC amplitude (left) and change in the synaptic delay (right) at PC synapses with long (> 500 μm) or short (< 500 μm) distance from the soma. **B, C**, Isopreterenol-caused change in axonal AP latency (B) and normalized AP amplitude at the PC soma and axons (C). Data were normalized to the average at -3 ~ -1 min.
